# Leveraging workflow control patterns in the domain of clinical practice guidelines

**DOI:** 10.1186/s12911-016-0253-z

**Published:** 2016-02-10

**Authors:** Katharina Kaiser, Mar Marcos

**Affiliations:** 1 Institute of Creative Media Technologies, St. Pölten University of Applied Sciences, St. Pölten, Austria; 2 Institute of Software Technology & Interactive Systems, Vienna University of Technology, Vienna, Austria; 3 Department of Computer Engineering and Science, Universitat Jaume I, Castellón, Spain

**Keywords:** Clinical practice guidelines, Computer-interpretable guidelines, Workflow control patterns, XSLT transformations

## Abstract

**Background:**

Clinical practice guidelines (CPGs) include recommendations describing appropriate care for the management of patients with a specific clinical condition. A number of representation languages have been developed to support executable CPGs, with associated authoring/editing tools. Even with tool assistance, authoring of CPG models is a labor-intensive task. We aim at facilitating the early stages of CPG modeling task. In this context, we propose to support the authoring of CPG models based on a set of suitable procedural patterns described in an implementation-independent notation that can be then semi-automatically transformed into one of the alternative executable CPG languages.

**Methods:**

We have started with the workflow control patterns which have been identified in the fields of workflow systems and business process management. We have analyzed the suitability of these patterns by means of a qualitative analysis of CPG texts. Following our analysis we have implemented a selection of workflow patterns in the Asbru and PROforma CPG languages. As implementation-independent notation for the description of patterns we have chosen BPMN 2.0. Finally, we have developed XSLT transformations to convert the BPMN 2.0 version of the patterns into the Asbru and PROforma languages.

**Results:**

We showed that although a significant number of workflow control patterns are suitable to describe CPG procedural knowledge, not all of them are applicable in the context of CPGs due to their focus on single-patient care. Moreover, CPGs may require additional patterns not included in the set of workflow control patterns. We also showed that nearly all the CPG-suitable patterns can be conveniently implemented in the Asbru and PROforma languages. Finally, we demonstrated that individual patterns can be semi-automatically transformed from a process specification in BPMN 2.0 to executable implementations in these languages.

**Conclusions:**

We propose a pattern and transformation-based approach for the development of CPG models. Such an approach can form the basis of a valid framework for the authoring of CPG models. The identification of adequate patterns and the implementation of transformations to convert patterns from a process specification into different executable implementations are the first necessary steps for our approach.

## Background

*Clinical practice guidelines* (CPGs) are defined as “systematically developed statements to assist practitioner and patient decisions about appropriate health care for specific circumstances” [1]. With this aim, CPGs include recommendations describing appropriate care (and, conversely, inappropriate care) for the management of patients with a specific clinical condition, such as diabetes or chronic heart failure. Consequently, an important part of CPG contents refers to the procedures to perform, ranging from concrete clinical actions (e.g., diagnostic and/or therapeutic interventions) to more or less complex combinations (e.g., sequences, choices) of actions. Despite some discrepancies, there is a wide consensus on the potential benefits of CPGs as regards the improvement of health-care. There is also consensus on the fact that the most effective way to deploy CPGs is through some kind of computerized tool, be it a reminder system or a more complex decision support system [2].

The description of CPGs in a computer executable form is a prerequisite for the development of computerized tools. The benefits of the use of executable CPGs in clinical settings are documented in the literature [3], and include improved guideline compliance and increased efficiency. A number of specialized representation languages have been developed to support executable CPGs [4–6]. These representation languages usually have associated authoring/editing tools to facilitate the construction of executable CPG models. Even with tool assistance, authoring of CPG models remains a complex and labor-intensive task that requires both clinical and technical skills.

It has been acknowledged for some time that the knowledge contained in CPGs is difficult to comprehend and formalize [7–12]. One reason identified by Patel et al. is that “CPGs can be semantically complex, often composed of elaborate collections of prescribed procedures with logical gaps or contradictions” [8]. On the other hand, CPG texts are aimed at clinicians with a large amount of specialized background knowledge. Thus, it is assumed that the reader posseses this background knowledge, which must be combined with the CPG information for a proper understanding. In this context, the combination of clinical and technical skills can prove fundamental. In an earlier study, Patel et al. analysed the CPG models developed by clinical staff alone, by technical staff alone, and by teams including both clinical and technical staff [7]. They concluded that the CPG models resulting from such teams were superior to the models developed by either clinical staff or technical staff alone.

### Motivation

We are concerned with facilitating the early stages of CPG modeling task. With this purpose, we aim to provide a series of procedural patterns in a notation that can be further refined and implemented in different target CPG representation languages. To make this possible, the main requirements we have set out for the patterns are on one hand their suitability for the expression of CPG procedural knowledge, and on the other hand their description using a neutral notation, to ensure the independence from the particular features of the different CPG languages. Applying patterns can reduce modeling time and enable stakeholders to communicate more precisely and in a less ambiguous way [13]. In turn, a faster modeling can serve to bring CPG recommendations almost immediately into clinical practice. Another important aspect is the implementation-independent specification of the control flow of these patterns. This would enable more flexibility in applying a CPG in different settings, by transforming the control specification into an implementation language.

For the patterns we have used as starting point the so-called *workflow control patterns,* 1 which are frequent task (and control) structures that have been identified in the fields of workflow systems and process modeling formalisms [14, 15]. Due to the similarities between Business Process Management (BPM) notations and CPG representation languages, these workflow patterns have been recently studied in the CPG literature, e.g. to analyze the expressivity of several representation languages [16, 17].

Note that although we are interested in leveraging workflow patterns for the description of CPG procedures, our focus is on CPG languages and systems. CPGs describe the processes to be performed for the management of an individual patient with a single clinical condition. A distinguishing feature of CPGs is that they target a care provider playing a specific role, usually the clinician [18]. By contrast, workflows in the clinical domain describe clinical processes not necessarily restricted to clinician’s work processes. Another difference is that workflows emphasise the administrative side of clinical processes at institutional level, focussing on scheduling visits, ordering laboratory tests, etc. in an effective and efficient way, for multiple patients (institution-centric view) [18]. On the other hand CPGs give more emphasis to the clinical knowledge that is required for clinical decision-making by the clinician, for a single patient (care provider-centric view). Several authors agree that both CPG and workflow systems fail to address important aspects of healthcare processes in general, when used individually [18, 19]. For instance, not surprisingly CPG systems are superior to workflow ones when it comes to represent the CPG knowledge supporting decision logic. For this reason, workflow systems would not be a good choice in our case, no matter how efficient available workflow engines are. Instead we focus our work on CPG languages and systems, which are understandably more appropriate for our purposes. Additionally, we concentrate on CPG systems which faithfully mirror CPGs (single-patient focus, and care provider-centric view). We do not consider other types of applications, e.g. auditing and cost analysis, as current CPGs do not provide information for such applications.

Given the difficulty and excessive workload associated to the authoring of CPG models, an approach that leverages a series of useful procedural patterns and supports their transformation into some of the alternative encodings would certainly facilitate the task. Consequently, the research questions that we investigate in this paper are the following: (1) are workflow control patterns suitable for the description of CPG procedural knowledge?, (2) can workflow patterns be adequately implemented in different executable CPG representation languages?, and (3) can the implementation of workflow patterns in alternative executable CPG languages be supported by means of semi-automated transformations?.

### Related work

Our work has features in common with various research efforts. In regard to the use of patterns to support the authoring of CPG models, there exist various works dealing with different kinds of patterns. Serban et al. [20] defined *linguistic patterns*, which can be used to formally represent the knowledge about medical actions contained in CPG texts. Furthermore, they combined these patterns with medical domain knowledge from existing medical thesauri (MeSH and NCI) into an ontology [21]. This ontology enables an easier formalization and maintenance of CPG models by allowing combining these patterns into more complex ones and by describing how they are linked to the textual representation. A similar approach was pursued by Kaiser et al. [22]. They defined *syntactic and semantic patterns* that are used to develop extraction rules to identify and extract actions and processes out of CPG texts. This approach was further developed by the definition of *semantic relation patterns* to automatically formalize actions in a CPG language [23]. The patterns are based on the UMLS Semantic Network and its semantic relations. Besides patterns at the text level, there are also pattern approaches at the algorithm level. Peleg and Tu [24] defined *design patterns* and developed visual templates that structure guideline steps restricted to the domain of screening algorithms. Nevertheless, *implementation patterns* are lacking, general enough and independent of the CPG domain and not restricted to types of procedures. Table 1 shows the main features of the different pattern types.

**Table 1:** Main features of the CPG patterns in the literature: input, output, transformation type, and goal

Pattern type	Input	Output	Transformation	Goal
*Linguistic patterns* [20]	Guideline text	Annotated guideline text	Automatic	Automatic identification of activity information, for mark-up. Applied as a pre-processing step in the manual development of CPG models.
*Syntactic and semantic patterns* [22]	Guideline text	Partial CPG model in an executable language	Semi-automatic	Semi-automatic generation of partial CPG models.
*Semantic relation patterns* [23]	Guideline text	Annotated guideline text	Automatic	Automatic identification of activity information. Can be applied in combination with syntactic and semantic patterns.
*Design patterns* [24]	Guideline text	CPG model in an executable language	Manual	Based on specific-purpose templates (screening and immunization) used in the manual development of CPG models.
*Implementation patterns*	CPG model in an intermediate notation	Partial CPG model in an executable language	Semi-automatic	Semi-automatic generation of partial CPG models using generic transformations (based on workflow patterns).

Concerning the intermediate and implementation-independent description of CPG models, there exist language proposals in the literature, notably GEM (Guideline Elements Model) [25] and MHB (Many-Headed Bridge) [9]. Both languages fall in the category of document-centric approaches [2], which are devised to produce a non-executable XML document with the relevant CPG fragments, starting from the original text. Both GEM and MHB are mark-up languages and encode recommendations as text, which does not allow automatic execution. As an illustration, see the following GEM example: <Recommendation>Treatment for extravasation of Vasopressors <Imperative>Contact primary service and stop protocol if patient has allergy to Phentolamine <Scope>Patient has allergy to Phentolamine</Scope><Directive>Contact primary service and stop protocol</Directive></Imperative></Recommendation>.

GEM has as strength the richness of elements for the description of CPG document details (e.g., authors, purpose, intended audience), but it has fallen short to map to executable CPG languages with the standard decision constructs [2]. In the case of MHB it has been shown that models can be evolved, not without difficulties, to the Asbru language. However, due to the Asbru-specific features of MHB (e.g., time elements are closely related to Asbru’s time annotations), one may question the feasibility of the correspondence to other executable CPG languages as it was initially planned.

Regarding the application of workflow patterns in the CPG domain, we want to point out the works by Mulyar et al. [16] and Grando et al. [17]. Mulyar et al. describe a pattern-based analysis of four CPG languages based on the implementability of workflow patterns in these languages. For the implementability aspect we apply nearly the same approach, except for the utilisation of CPG execution engines in our case (see below). However, Mulyar et al. consider the whole set of workflow patterns, whereas we only analyse those workflow patterns that are deemed relevant for the description of CPG procedural knowledge, on the basis of a prior suitability study. As result they conclude that BPM languages are superior to CPG ones, since the former provide a better support for the whole set of workflow patterns. Grando et al. provide a formal method to demonstrate the implementability of workflow patterns in a given language. The method is based on Coloured Petri Nets (CPNs) and the notions of congruence and bisimilarity from pi-calculus, and supposes the description of both the pattern and the implementation thereof in terms of CPNs. Grando et al. also provide an illustration of the method using three workflow patterns. The use of CPNs is a strong requirement since the description of patterns and implementations as CPNs might not be trivial. Note that the works by Mulyar et al. and Grando et al. do not consider the suitability of workflow patterns, which we emphasise.

The transformation-based approach we propose for the authoring of CPG models presupposes a gradual conversion process. In the literature, the DeGeL architecture [26] and the tools of Protocure II project [27] are representative of platforms dealing with multiple CPG models with increasing formalization levels. More recently, the platform by Pérez et al. [28, 29] uses a similar approach, in this case implemented using Model-Driven Development techniques. However, none of these frameworks considers semi-automated transformations during early development stages of CPG models. For instance, in the Protocure II project the semi-automated transformations are applied once the formalised Asbru model has been manually completed, to obtain the corresponding model-checking representation, for verification purposes. In the case of the platform by Pérez et al. the transformations are applied to CPGs modelled as UML statecharts, also to obtain a model-checking representation. In our view neither Asbru nor UML statecharts are adequate for the early development stages, where comprehensibility should prevail.

## Methods

We are concerned with the early stages of CPG model development, in particular with the modeling of procedural knowledge fragments in the CPG text. In this context, we argue for supporting the authoring of CPG models using a set of suitable procedural patterns described in an intermediate and implementation-independent notation that can be then semi-automatically transformed into one of the alternative executable CPG languages. Following our approach, the authoring of executable CPG models will involve as a first step (1) the identification of the procedural fragments in the CPG text. Then, (2) a first modeling of these fragments will be carried out in some implementation-independent notation, using common procedural patterns as a guide. Afterwards, (3) the models from the previous step will be converted into some executable CPG language, using automatic transformations where possible. Lastly, (4) these partial executable models will be combined and fine-tuned to complete the final executable CPG model.

The description of patterns, and ultimately CPGs, in an implementation-independent notation can serve as a basis for the subsequent implementation in different executable CPG languages, provided that appropriate transformation algorithms are developed. With such transformations, the efforts invested in modelling the CPG in the implementation-independent notation can be leveraged for the implementation in different CPG languages. This constitutes an advance since models can be potentially reused in different implementation settings, thus saving modelling time and resources.

We have made developments towards our pattern and transformation-based approach. For the procedural patterns we have started from the workflow patterns by Russell et al., restricted to a selection of patterns deemed suitable for the procedures that appear in CPGs. The suitability of patterns has been determined through a qualitative analysis of a small set of CPG texts. Additionally, the selected workflow patterns have been implemented in two different executable CPG languages, namely Asbru [30] and PROforma [31]. As implementation-independent notation we have chosen the Business Process Model and Notation (BPMN) 2.0 [32], which is the latest Object Management Group’s (OMG) standard for BPM and for which workflow patterns’ descriptions can be found in the literature [33]. Our choice matches that of the latest literature, such as the work by Kossak et al. [34]. We fully agree with the arguments that these authors provide to support the choice of BPMN, namely: it is an international standard issued by a well-established group with a strong foundation in the industry (OMG); it has already gone through a maturing process; and it has been widely adopted. Furthermore, there is evidence that BPMN is suitable for the medical domain, e.g. for the development of clinical pathways [35]. On the negative side, the authors report the high amounts of manpower and time required for BPMN modelling. Despite this, they conclude that the use of a modelling language such as BPMN could be a reasonable approach for the development of clinical information systems. Finally, we have developed XSLT (XSL Transformations, where XSL stands for Extensible Stylesheet Language) transformations of selected workflow patterns, to convert the BPMN 2.0 notation into the Asbru [30] and PROforma [31] languages. Notice that other choices would be possible in our approach (e.g. other languages, and even other patterns).

### Analysis of workflow control patterns for CPGs

One of the requirements we have set for the procedural patterns is that they are suitable for the description of CPG procedural knowledge, in the sense that there exists a correspondence between the task structures that the patterns describe and the kinds of procedures that can be found in CPGs. Based on this idea, we have analyzed Russell et al. workflow patterns to determine whether the task (and control) structures they embody have a counterpart in the diagnostic and/or therapeutic procedures that usually appear in CPGs. This is important because these patterns were originally identified as generic, recurring constructs in the fields of workflow systems and BPM [15], and hence some of them might not be relevant to the CPG domain.

The authors performed a qualitative suitability analysis. Both authors are computer scientists with a strong research record on CPG modeling. The qualitative analysis was based on a sample of 8 real-world CPGs taken from different medical specialties, namely Cardiology, Emergency Medicine, Obstetrics, Oncology, Pediatrics, and Pulmonology. The sample set was chosen from CPGs that the researchers had previously used in their work. It includes CPGs representative of typical processes (such as sequences, choices, iterations, etc.) according to the researchers’ experience. The set also includes other CPGs with additional features, e.g. CPGs from Emergency Medicine were included in the sample set, because in this specialty different tasks are often executed in parallel under limiting time constraints. Table 2 lists different features of the CPG sample. These include the specialty, intended users, category, and developer of the CPGs. Information on the structure of the CPG document and on the availability of flowcharts is also shown. As can be seen in Table 2, the characteristics of the CPGs are diverse.

**Table 2:** Main features of the CPG sample used, describing the specialty, intended users, guideline category and developers, as well as information about the structure of the CPG document

Title of the guideline	Main clinical specialty	Intended users	Guideline category	Guideline developer(s)	Pages	Document features	Flowchart
Induction of labour [54]	Obstetrics and Gynecology	Advanced Practice	Counseling	National Government Agency	32	Well-structured text with clearly marked recommendations. No grading schemes for evidence and recommendations are used	Algorithm available as external resource
		Nurses	Evaluation				
		Allied Health Personnel	Management				
		Health Care Providers	Prevention				
		Nurses	Risk Assessment				
		Patients					
		Physician Assistants					
		Physicians					
		Public Health Departments					
SPREAD—Stroke Prevention and Educational Awareness Diffusion [55]	Emergency Medicine	Advanced Practice Nurses	Diagnosis Evaluation	National Medical Specialty Society	53	Well-structured text with clearly marked recommendations. Uses both evidence levels and recommendation grades	Not available
		Allied Health Personnel	Management				
		Emergency Medical Technicians/Paramedics	Prevention				
		Health Care Providers	Risk Assessment				
		Health Plans	Treatment				
		Hospitals					
		Managed Care Organizations					
		Nurses					
		Pharmacists					
		Physician Assistants					
		Physicians					
Guideline for the Treatment of Breast Carcinoma [56]	Oncology	Advanced Practice Nurses	Diagnosis	National Disease Specific Society	117	Well-structured text with clearly marked recommendations. All the relevant studies are discussed. Uses both evidence levels and recommendation grades	Not available
			Management				
		Allied Health Personnel	Treatment				
		Health Care Providers	Rehabilitation				
		Nurses					
		Physician Assistants					
		Physicians					
		Psychologists					
Evidence-based care guideline for Fever of Uncertain Source in infants 60 days of age or less [57]	Pediatrics	Advanced Practice Nurses	Diagnosis	Hospital/Medical Center	14	Well-structured text with clearly marked recommendations. Uses evidence levels but not recommendation grades	Algorithm available within the document
			Evaluation				
		Health Care Providers	Management				
		Nurses	Risk Assessment				
		Patients	Treatment				
		Physician Assistants					
		Physicians					
Diagnosis and treatment of chest pain and acute coronary syndrome (ACS) [58]	Cardiology	Advanced Practice Nurses	Diagnosis	National Nonprofit Organization	91	Document with an algorithm as main element, including algorithm annotations. Uses evidence levels but not recommendation grades	Algorithms available within the document
			Evaluation				
		Allied Health Personnel	Management				
		Emergency Medical Technicians/Paramedics	Rehabilitation				
		Health Care Providers	Risk Assessment				
		Health Plans	Screening				
		Hospitals	Treatment				
		Managed Care Organizations					
		Nurses					
		Pharmacists					
		Physician Assistants					
		Physicians					
Management of Hyperbilirubinemia in the Newborn Infant 35 or More Weeks of Gestation [59]	Pediatrics	Advanced Practice Nurses	Diagnosis	National Medical Specialty Society	18	Well-structured text with clearly marked recommendations. Uses evidence levels but not recommendation grades	Algorithm available within the document
		Allied Health Personnel	Management				
		Dietitians	Treatment				
		Hospitals					
		Nurses					
		Physician Assistants					
		Physicians					
Global strategy for the diagnosis, management, and prevention of chronic obstructive pulmonary disease: GOLD executive summary [60]	Pulmonary Medicine	Advanced Practice Nurses	Counseling	International Disease Specific Society	24	Non structured text including several tables; recommendations are not marked. Uses evidence levels but not recommendation grades	Not available
			Diagnosis				
		Allied Health Personnel	Evaluation				
		Nurses	Management				
		Physician Assistants	Prevention				
		Physicians	Risk Assessment				
		Public Health Departments	Treatment				
		Respiratory Care Practitioners					
Guidelines for the diagnosis and treatment of chronic heart failure: executive summary [61]	Cardiology	Advanced Practice Nurses	Diagnosis	European Medical Specialty Society	26	Non structured text including several tables; recommendations are not marked. Uses both evidence levels and recommendation grades	An algorithm for diagnosis available within the document
			Evaluation				
		Health Care Providers	Management				
		Nurses	Risk Assessment				
		Pharmacists	Treatment				
		Physician Assistants					
		Physicians					

For each workflow pattern, the researchers have thoroughly studied the associated documentation (including the rationale and intended operational context) and then they have searched the CPG texts for fragments amenable to be described using the pattern. Pattern examples have been individually obtained by each of the two researchers. As an illustration, the text fragment “A before B” can be regarded as a sequence. The search is guided by the CPG text and does not consider activities at levels of granularity different from what the CPG text describes explicitly. The latter is based on several considerations. A fundamental one is that CPGs, and consequently CPG systems, are not expected to describe the full details of the procedures for which physicians have been trained. As result, the common practice is setting the modelling boundaries in standard clinical procedures, concretely those that usually appear in medical terminologies. Our work in confined to this common practice.

According to the nature of CPG processes, a number of criteria were fixed in advance as to what control features could be considered useful or relevant (see section “Analysis of workflow control patterns” for more details). E.g., these include basic control patterns, but not the ones related to multiple execution threads, among others. Thanks to these criteria, there was little disagreement on the pattern suitability between the two researchers (Cohen’s kappa coefficient is 98 %). Afterwards, the examples have been jointly discussed by the two researchers, and an agreement has been reached on the applicability of each of the patterns.

Our analysis is influenced by a sample selection bias, due to the reduced size of the CPG sample and to the fact that this sample was selected among the CPGs with which the authors were familiar. To avoid the possible negative implications of this bias, no pattern has been considered unsuitable on the basis of the lack of supporting examples in the CPG sample. Another possible source of bias stems from the subjective nature of our analysis. Qualitative studies have been criticised of being highly subjective, largely because the researcher is the instrument for both data collection and interpretation [36]. However, this does not necessarily imply that a qualitative analysis should rely exclusively on the judgement and criteria of one researcher. For instance, the notes and results of the researcher can be reviewed and critiqued by peers as a check for personal bias [37]. The method we describe in this section is based on this idea. The same holds for the method we describe in section “Transformations for the implementation of workflow control patterns”.

### Implementation of workflow control patterns for CPGs

One of our objectives is providing an implementation of selected workflow patterns in different target CPG representation languages. For this purpose, we have chosen the Asbru [30] and PROforma [31] representation languages. The main criteria for this choice were, on one hand, the availability of a non-commercial execution tool with some sort of testing functionality that allows the validation of implementations, and, on the other hand, the existence of recent activity and/or developments in relation to the language. Other widely cited languages, such as EON [38], GLIF [39] and SAGE [40], have not been considered because they are part of projects that are no longer active (and/or the associated execution tools are no longer maintained).

We have concentrated on the implementation of the workflow patterns that are relevant from the perspective of CPG processes (see sections “Analysis of workflow control patterns for CPGs” and “Analysis of workflow control patterns”). The implementation has been carried out by the two authors of this article, who have advanced knowledge and skills in the Asbru and PROforma languages. One of them has implemented the patterns in Asbru, and the other one in PROforma. The pattern implementations have been tested thoroughly to ensure that they correspond to their intended behavior according to Russell et al. descriptions. Afterwards, all the implementations (Asbru and PROforma ones) have been independently assessed by the two researchers. Here again, the discrepancies between the two researchers were minimal (Cohen’s kappa coefficient is also 98 %). Thus, an agreement for each particular implementation could be easily reached after discussion between the researchers.

### Transformations for the implementation of workflow control patterns

In addition to an actual implementation of the selected workflow patterns in the Asbru and PROforma languages, we are concerned with providing a description of the same patterns in an implementation-independent notation together with a series of semi-automated transformations to obtain executable counterparts in the previous CPG languages. The implementation-independent notation that we have chosen is the OMG standard BPMN 2.0 [32]. The main reason for this choice is that BPMN has been widely adopted as a graphical notation for BPM, being used by humans to design, visualize and manage business processes ranging from workflows to automated business processes. Furthermore, the XML syntax (and vocabulary) for BPMN 2.0 makes it possible to exploit XML technology, notably the XSLT language [41].

To obtain our BPMN 2.0 models, we have used as starting point the BPMN description of workflow patterns in the literature. For the definition of XSLT transformation rules, both the source BPMN 2.0 description and the target (Asbru or PROforma) implementation of each pattern must be taken into account. Additionally, this requires analyzing how BPMN 2.0 language constructs map to Asbru and PROforma ones. Note that the definition of XSLT transformations is far from straightforward in some cases, since there is not always a 1:1 relationship between the elements of the notations. For instance, BPMN has specific elements for both diverging and converging gateways, but neither Asbru nor PROforma have such elements. Also, PROforma has elements for decisions, which do not exist in BPMN. These differences make the development of transformation rules a difficult task. Although the relation between the elements of the source and target notations is not always 1:1, the source and target representations of the patterns are known in all cases. The development of the XSLT transformations has been shared between the two authors, each devoted to one of the languages. All the transformations have been tested, and the resulting models have been validated using the Asbru and PROforma tools.

## Results

### Analysis of workflow control patterns

Table 3 shows the consensual results of the analysis, together with an explanation of the suitability (or non-suitability) of each pattern. The table also lists examples of workflow patterns found in the studied CPGs. Bold rows present the workflow control patterns that were finally deemed suitable. Next, we detail the most important findings of our analysis. The labels in the second column should be read as follows: “no” indicates that there are strong reasons to dismiss the applicability of the pattern in the CPG domain; “yes” indicates that examples of the pattern have been found in the sample CPGs; otherwise, “unknown” means that no examples were found in the CPGs.

**Table 3:** Suitability of workflow control patterns for CPGs

Pattern	Suitable	Explanation	Example
**1. Sequence**	**yes**	**-**	***Stabilize on tocolytics before transfer mother to appropriate level of care if possible*** **[** 27 **]**
**2. Parallel split (AND split)**	**yes**	**-**	***The maternal pulse should be felt simultaneously to differentiate between maternal and fetal heart rate*** **[** 27 **]**
**3. Synchronization (AND join)**	**yes**	**explicit/implicit in a sequencing after a parallel split**	***Zidovudin for one hour and single dose of Nevirapine 30 min before the skin incision. Afterwards give Retrovir until birth*** **[** 27 **].**
**4. Exclusive choice (XOR split)**	**yes**	**-**	***If diastolic blood pressure >140 mmHg occurs on two readings 5 min apart, then start a continuous IV infusion of an antihypertensive agent*** **[** 28 **]**
**5. Simple merge (XOR join)**	**yes**	**explicit/implicit in a sequencing after an exclusive choice**	***… locoregional postoperative radiotherapy (after BCT or MRM)*** **[** 29 **]**
**6. Multi-choice (OR split)**	**yes**	**-**	***Add regular treatment with one or more bronchodilators*** **[** 30 **].**
**7. Structured synchronizing merge (OR join)**	**yes**	**explicit/implicit in a sequencing after a multi-choice**	**(see example above)**
8. Multi-merge	no	multiple activation of a task only in structured loops	
**9. Structured discriminator**	**unknown**	**synchronization of 2 or more branches waiting for the first incoming branch has not been found in guidelines**	
10. Arbitrary cycles	no	multiple activation of a task only in structured loops	
**11. Implicit termination**	**yes**	**by definition**	
12. MIs without synchronization	no	no multiple, concurrent instances of a task	
13. MIs with a priori design-time knowledge	no		
14. MIs with a priori run-time knowledge	no		
15. MIs without a priori run-time knowledge	no		
**16. Deferred choice**	**yes**	**-**	***Surgery to reduce tumour load. It is unclear whether limited or radical surgery is better*** **[** 29 **]** *.*
**17. Interleaved parallel routing**	**unknown**	**WCP-40 with the possibility of adding partial ordering constraints has not been found in sample guidelines**	
**18. Milestone (deadline)**	**yes**	**WITH or WITHOUT activity disablement beyond the milestone**	***In patients hypoxemic during a COPD exacerbation, arterial blood gases and/or pulse oximetry should be evaluated prior to hospital discharge*** **(WITH) [** 30 **]** ***ICD implantation is reasonable in selected patients with LVEF < 30-35 %, not within 40 days of a myocardial infarction, on optimal background therapy …*** **(WITHOUT) [** 31 **]**
**19. Cancel activity**	**yes**		***If renal function deteriorates substantially, stop treatment*** **[** 31 **]**
**20. Cancel case**	**yes**	**-**	***Patients with apparent exacerbations of COPD that do not respond to treatment should be re-evaluated for other medical conditions*** **[** 30 **]**
**21. Structured loop**	**yes**	**typically in follow-up activities**	***Follow-up: first year: once every three months; second year: once every six months; subsequently: annually*** **[** 29 **].**
22. Recursion	no	multiple activation of a task only in structured loops	
**23. Transient trigger**	**yes**	**interpreted as triggers to be acted on immediately**	***Administer controlled oxygen therapy and repeat arterial blood gas measurement after 30–60 min*** **[** 30 **].**
**24. Persistent trigger**	**yes**	**interpreted as triggers to be acted on either immediately or at some future time**	***Spirometry should be performed if there is a substantial increase in symptoms or a complication*** **[** 30 **]** *.*
**25. Cancel region**	**yes**	-	***Discontinue drugs that may lower heart rate in presence of bradycardia*** **[** 31 **].**
26. Cancel MI activity	no	no multiple, concurrent instances of a task	
27. Complete MI activity	no		
28. Blocking discriminator	no		
29. Cancelling discriminator	no	no concurrent execution of tasks within cancelation	
**30. Structured partial join**	**unknown**	**synchronization of 2 or more branches waiting for the first N incoming ones has not been found in guidelines**	
31. Blocking partial join	no	no multiple execution threads	
32. Cancelling partial join	no	no concurrent execution of tasks with cancelation	
33. Generalized AND join	no	no multiple execution threads	
34. Static partial join for MIs	no	no multiple, concurrent instances of a task	
35. Cancelling partial join for MIs	no		
36. Dynamic partial join for MIs	no		
37. Acyclic synchronizing merge	no	useful for non-structured models only	
38. General synchronizing merge	no	multiple activation of a task only in structured loops	
**39. Critical section**	**yes**	**-**	***The simultaneous administration of radiotherapy and chemotherapy … is discouraged*** **[** 29 **]**
**40. Interleaved routing**	**yes**	**-**	***Based on ‘expected survival benefit’ no statement can be made. Regarding the optimum sequence of radiotherapy and chemotherapy … The simultaneous administration of radiotherapy and chemotherapy … is discouraged*** **[** 29 **]**
41. Thread merge	no	no multiple execution threads	
42. Thread split	no		
**43. Explicit termination**	**yes**	**-**	***Discharge with planned follow-up*** **[** 32 **]**

Legend of ‘Suitable’ column: ‘yes’ indicates that the pattern has been found in sample CPGs; ‘unknown’ that it has not been found in sample CPGs; and ‘no’ that there exist strong reasons to dismiss the applicability of the pattern in the CPG domain. Bold rows present the workflow control patterns that were finally deemed suitable

In the analysis we have taken into account the nature of the processes in CPGs. In particular we have taken into account the fact that CPGs address the management of individual patients/cases, and that in general CPGs ultimately refer to unstructured human processes that are not executable. Considering this we have directly ruled out the multiple instance (MI) patterns (#12-#15, #26, #27, and #34-#36), which involve multiple instances of an activity/task running concurrently, as well as the patterns related to multiple execution threads (#28, #31, #33, #41, and #42). The use of these patterns, which are closely related to executable processes, is of limited interest in the context of CPGs.

Moreover, we have taken into account that CPG processes are usually formulated in natural language. In this context the standard formulation for iterative tasks is through sentences such as “REPEAT x EVERY t”, i.e. using the pattern *“structured loop”*. Consequently, we have excluded other patterns involving loops or multiple activations of a task, including recursion (#8, #10, #22, and #38). Finally, we have dismissed the patterns intended to be used in the context of non-structured process models (#37, and again #38). Roughly speaking, a structured model is one in which every split element (e.g. AND split) has a matching join element of the same type, and in which all split-join pairs are properly nested [42, 43]. Since CPG processes are formulated in natural language, non-structuredness does not appear to be an essential nor useful feature for CPGs.

Apart from the above, we have identified a few patterns of unknown applicability, due to the lack of supporting examples in the sample CPGs. Among them we can cite the patterns *“structured discriminator”* (#9), also known as 1-out-of-M join, and *“structured partial join”* (#30), which is an N-out-of-M join. In both cases there is a synchronization of several incoming branches when a certain number of them (respectively, 1 or N) have completed, with the nuance that completion of additional incoming branches is permitted but has no effect. The versions of these patterns which operate terminating pending incoming branches after synchronization, namely *“cancelling discriminator”* and *“cancelling partial join”* (#29, and #32), deserve a separate comment. They involve the concurrent execution of a series of activities that are actually alternative attempts to expedite a task, and that therefore can be terminated once one of the attempts has successfully completed. Inherently, CPGs make a choice among alternative courses of action for a patient, taking into account the costs and health outcomes associated with the alternatives [44]. This suggests that the patterns for concurrent execution with cancellation after synchronization would not be needed in the CPG domain (and hence they have been labeled as “no” in Table 3).

#### Summary

Our suitability analysis has served to identify the workflow patterns that are relevant to the CPG domain (see rows in bold text in Table 3). These include all patterns except those related to MIs, multiple activations of tasks, non-structured loops, non-structured processes, or concurrent tasks with cancellation (all labeled as “no” in Table 3). Those patterns of unknown applicability (labeled as “unknown” in Table 3), for which we found no examples in the sample CPGs, have been also considered as relevant. Finally, as a by-product of the analysis we have identified a number of patterns of potential interest in the CPG domain.

Thus, we have shown that workflow control patterns by Russell et al. can be used to describe CPG procedural knowledge. Nevertheless, our analysis has revealed that many of the patterns provided are not essential to describe CPG procedural knowledge. CPGs are developed to provide decision-support for a single patient rather than for describing the workflow for a care provider handling multiple patients. This might explain that only about 51 % of the workflow control patterns are required to describe CPGs.

### Implementation of workflow control patterns for CPGs

Table 4 shows the agreed results on the implementability of the patterns in Asbru and PROforma. These results should be read as follows: “+” indicates that the pattern is directly implementable via dedicated language constructs; “+/−” indicates that the pattern can be implemented indirectly using other constructs; and “-” means that the pattern cannot be implemented due to the peculiarities of the language.

**Table 4:** Implementation of selected workflow control patterns in the Asbru and PROforma languages

Pattern	Implementable	
	Asbru	PROforma
1. Sequence	+	+
2. Parallel split (AND split)	+	+
3. Synchronization (AND join)	+	+
4. Exclusive choice (XOR split)	+	+
5. Simple merge (XOR join)	+	+
6. Multi-choice (OR split)	+	+
7. Structured synchronizing merge (OR join)	+	+
9. Structured discriminator	+	+
11. Implicit termination	+	+
16. Deferred choice	+	+
17. Interleaved parallel routing	+	-
18. Milestone (deadline)	+/−	+
19. Cancel activity	+	+
20. Cancel case	+	+
21. Structured loop	+	+
23. Transient trigger	-	+
24. Persistent trigger	+/−	+
25. Cancel region	+/−	+
30. Structured partial join	+	+
39. Critical section	+	-
40. Interleaved routing	+	-
43. Explicit termination	+	+

Legend of ‘Implementable’ column: ‘+’ indicates that the pattern is directly implementable; ‘**+/−**’that it is not directly implementable; and ‘-’that it is not implementable

Implementations to model a pattern have been labeled with “+/−”when they contain a significant number of additional variables. These variables serve to mimic either triggers or milestones that are used in conditions to manage the execution of the plans. Such implementations are valid but make the resulting model excessively complicated, in our view. On the other hand, the label “-”refers to the non-implementability of the pattern in its full meaning, considering the peculiarities of the language. As described below, some sort of implementation might still be possible, although disregarding the full meaning/rationale of the pattern. We do not consider that such implementations are valid.

Our results differ slightly from those obtained by Mulyar et al., concerning the implementability of the patterns *“cancel region”* and *“explicit termination”*. We found that “cancel region” is implementable in both Asbru and PROforma, by means of a plan grouping the activities to cancel with a suitable abort condition. “Explicit termination” is also implementable in both languages. In this case an appropriate condition (complete condition in the case of Asbru and termination condition in PROforma) referring to the designated state must be added to the top-level plan. Our results also differ from those presented by Grando et al., concerning the implementability of the pattern *“simple merge”*. According to our results this pattern can be modelled both in Asbru and PROforma, however Grando et al. conclude that it cannot be modelled in PROforma. This may be due to a misinterpretation of the pattern, which in principle corresponds to an XOR (exclusive) join, resulting in an inaccurate CPN model.

In the rest of the section we report on our implementation of the workflow control patterns. For space reasons, our account is limited to one essential pattern in the CPG domain, which is *“exclusive choice”*, plus other two patterns to illustrate specific features of the PROforma and Asbru languages, namely *“persistent trigger”* and *“interleaved routing”*. The *“exclusive choice”* pattern is presented together with the *“simple merge”* one for improved understanding, since this combination constitutes a typical usage scheme.

#### Implementation of workflow control pattern combination *“exclusive choice - simple merge”* (#4-#5)

The *“exclusive choice”* pattern addresses the need for directing the flow of control to a particular task, depending on a logical condition which is typically based on the value of specific data items or on the results of a user decision. In the medical domain, this pattern allows enabling a particular action under certain clinical circumstances. It also allows the choice among alternative courses of action that is common in CPGs. An example, extracted from a stroke prevention and management CPG, is: *“If diastolic blood pressure >140 mmHg occurs on two readings 5 min apart, then start a continuous IV infusion of an antihypertensive agent”*.

As mentioned before, an *“exclusive choice”* is usually followed by a *“simple merge”* that joins the branches directing the flow of control to the subsequent tasks. All CPG languages support this pattern combination in a fairly direct way, and so do Asbru and PROforma.

##### Asbru

**Fig. 1:**
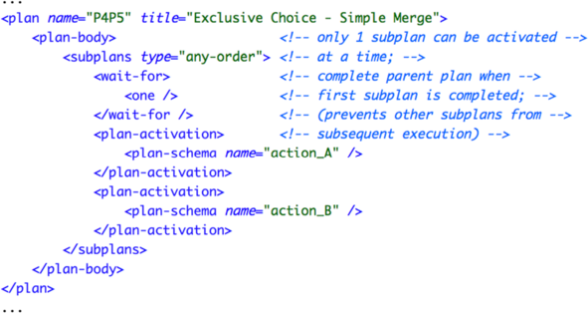
Implementation of pattern combination “exclusive choice—simple merge (#4-#5) in Asbru

In Asbru there are two ways to model this pattern combination. On the one hand, there is the procedural approach using the if-then-else construct. On the other hand, in the declarative approach alternatives are modeled as subplans, where all subplans are associated with mutually-exclusive filter conditions that force the execution of only one subplan. We have applied the declarative approach, which is clearer when there exist multiple alternatives to choose among. As soon as one subplan is executed the parent plan completes preventing the execution of the remaining alternatives. In the declarative approach this is accomplished by using “any-order” subplans in combination with the expression wait-for="one". Figure 1 shows the implementation of this pattern combination, with parent plan exclusive_choice having action_A and action_B subplans as alternatives.

##### PROforma

**Fig. 2:**
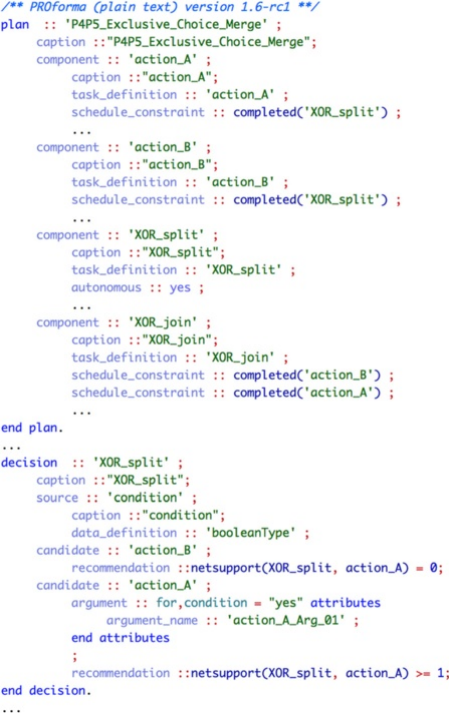
Implementation of pattern combination “exclusive choice—simple merge” (#4-#5) in PROforma

Our implementation of the *“exclusive choice—simple merge”* pattern combination makes use of the decision task, which is one of the four main types of tasks in PROforma. A decision requires the specification of both the candidates or possible results of the decision, and the arguments for and/or against them. The choice mode (single vs. multiple candidate selection) must be specified as well. Figure 2 shows the PROforma implementation of an exclusive choice with two alternative actions, named action_A and action_B. The choice is implemented in the decision XOR_split, which is a single selection choice with two candidates, one for each one of the alternative actions. The candidate action_A is recommended when a certain condition holds, using an appropriate argument with an expression condition="yes", whilst the candidate action_B is recommended in case this argument is not applicable.

#### Implementation of workflow control pattern *“persistent trigger”* (#24)

The pattern *“persistent trigger”* allows proceeding with a task (or initiating it) in response to a signal from another task in the process or from the external environment. The signal/trigger is persistent in the sense that it remains active in the execution context. In the CPG domain, this pattern enables e.g. the execution of tasks to deal with special patient circumstances. One example, drawn from the follow-up part of a CPG for chronic obstructive pulmonary disease, is: *“Spirometry should be performed if there is a substantial increase in symptoms or a complication”*. In this case the signal would come from the anamnesis and/or physical examination during the patient encounter.

The support for triggers varies considerably from language to language. Thus, although the implementation of this pattern in PROforma is almost straightforward, it can only be done in Asbru through programming workarounds (see +/− label in Table 3). In the rest of the section we focus on the PROforma implementation.

##### PROforma

**Fig. 3:**
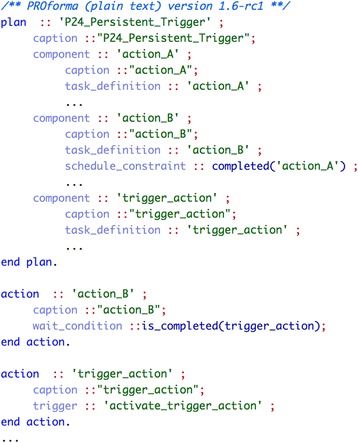
Implementation of pattern “persistent trigger” (#24) in PROforma

Our implementation of the pattern *“persistent trigger”* uses a state trigger in the target task. The expression in a state trigger may refer either to some data item or to the state of a task. We have opted for an expression referring to the completion of another (triggering) task. Optionally, an event trigger can be used to initiate the triggering task, representing a signal from the external environment. Figure 3 shows the PROforma implementation of a sequence of two actions, action_A and action_B, where the second one additionally depends on the completion of a triggering task, trigger_action. This is specified by means of a state trigger (see the wait_condition attribute).

#### Implementation of workflow control pattern “*interleaved routing*” (#40)

The pattern *“interleaved routing”* allows the execution of a series of tasks in any sequential order, i.e. one task after the other but without specifying a concrete sequence of tasks. This pattern is ubiquitous in the CPG domain, e.g. a series of diagnostic tests are often requested and the actual ordering is not relevant. It is also frequent to indicate that the actual ordering of two or more procedures is left at the choice of the clinician. A good example of the latter case, taken from an Oncology CPG, is: *“Based on ‘expected survival benefit’ no statement can be made regarding the optimum sequence of radiotherapy and chemotherapy. … The simultaneous administration of radiotherapy and chemotherapy (particularly for anthracycline-containing regimens) is discouraged”*.

In PROforma tasks are executed in parallel by default, unless scheduling constraints are specified. Therefore, the implementation of the *“interleaved routing”* pattern would require an enumeration of all possible permutations of tasks, hence contradicting the rationale of the pattern. In contrast, Asbru has a dedicated construct for this pattern as we describe below.

##### Asbru

**Fig. 4:**
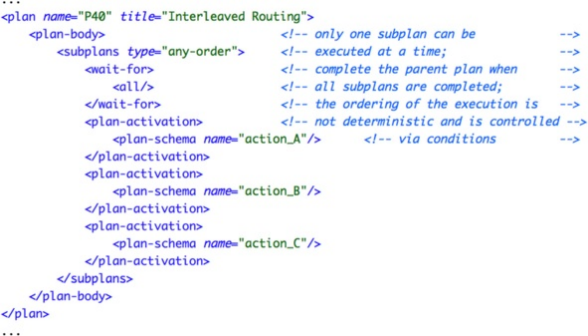
Implementation of pattern “interleaved routing” (#40) in Asbru

In Asbru the pattern is implemented using “any-order” subplans, similarly to pattern #4-#5. Thereby, only one of the defined subplans can be executed at a time and each subplan is executed exactly once. In contrast with pattern #4-#5, the parent plan needs to complete as soon as all its subplans are completed, which is accomplished by the expression wait-for="all". Figure 4 shows the implementation of the “interleaved routing” pattern with three subplans, named action_A, action_B, and action_C.

#### Summary

Following our suitability analysis we have implemented the selected workflow patterns in the Asbru and PROforma languages. To ensure that the implementations accurately correspond to the intended semantics of workflow patterns as shown by Russell et al. [15, 45], they were tested using the Asbru Interpreter [46] and the Tallis Suite [47], respectively. The tests consisted in making sure that the execution traces obtained by executing the implementations were consistent with the execution traces documented in the literature. In other words, we have approached the implementability analysis similarly to Grando et al., although not formally. Overall we may say that the capabilities of Asbru and PROforma to implement the selected patterns is significant, with the exception of trigger patterns in Asbru and interleaved-routing ones in PROforma (see Table 4 for details). Although there are patterns that cannot be directly implemented in each of these languages, it would be ultimately possible to model them through programming workarounds [48].

### Transformations for the implementation of workflow control patterns

Next we outline both the BPMN 2.0 descriptions of selected workflow patterns and the XSLT transformations to semi-automatically generate their Asbru and/or PROforma counterparts. For the sake of brevity, our account is limited to the same pattern combinations/patterns described in section “Implementation of workflow control patterns for CPGs”.

#### Transformations for workflow control pattern combination *“exclusive choice – simple merge”* (#4-#5)

**Fig. 5:**
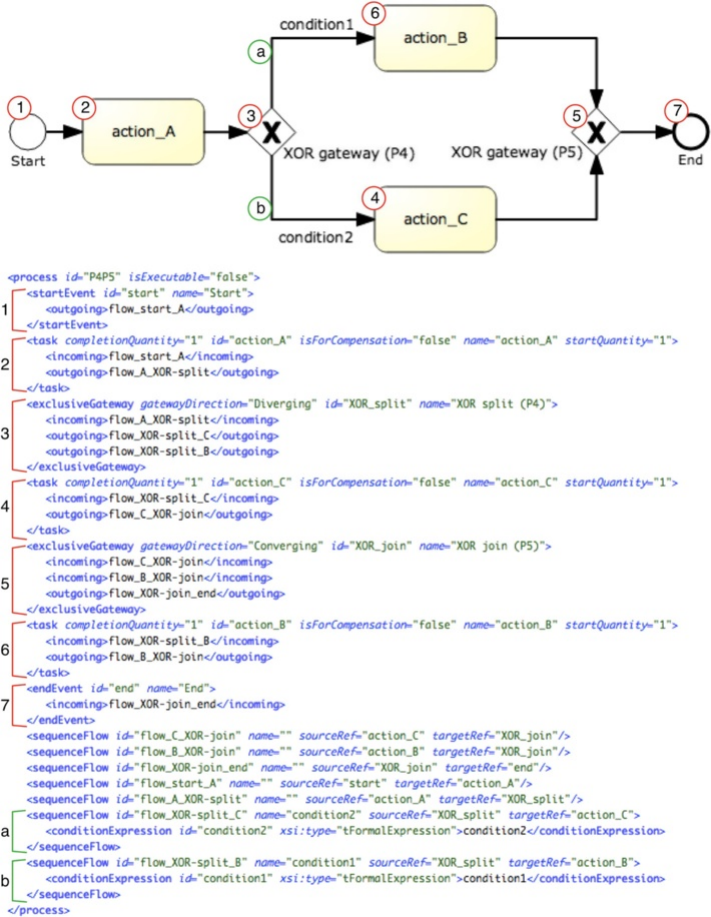
Description of the pattern combination “exclusive choice—simple merge” (#4-#5) in BPMN 2.0

Figure 5 shows the BPMN description of the pattern combination *“exclusive choice—simple merge”*. As explained before, an *“exclusive choice”* is typically followed by a *“simple merge”* that joins the diverging branches prior to the subsequent tasks. The pattern starts with an exclusive gateway (label 3 in the figure) diverging to two or more tasks (labels 4 and 6) that afterwards converge into another exclusive gateway (label 5). The pattern ends with the latter converging gateway. For selecting the task after the diverging gateway mutually exclusive conditions are specified in the outgoing arcs (see labels *a* and *b* in Fig. 5).

##### Asbru

**Fig. 6:**
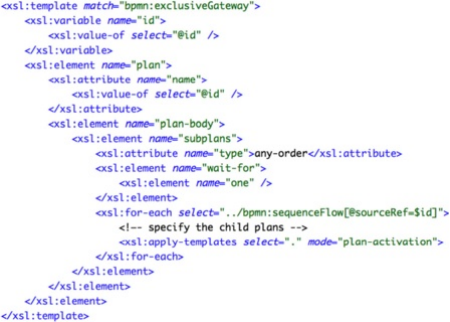
Fragment of the transformations for generating the Asbru code for the *“exclusive choice—simple merge”* pattern combination: generation of Asbru plan for the elements enclosed in gateways

For the transformation into Asbru, the diverging exclusive gateway (see label 3 in the figure) and every subsequent task (labels 4 and 6) have been transformed into Asbru plans. Then, the plan-body of the gateway plan has been completed with an element subplans of type “any-order” and with the additional constraint wait-for="one", to allow the activation of only one subplan. Every task after the gateway has been then referenced as a subplan by means of a plan activation. The plans for these tasks contain appropriate filter-precondition elements specifying the conditions that are obtained from the corresponding sequenceFlow elements (labels *a* and *b*). Figure 6 shows an excerpt of the transformations for generating the block for the exclusive choice containing the elements enclosed in gateways.

##### PROforma

To obtain an equivalent PROforma plan, every BPMN start/end event, exclusive gateway, and task has been transformed into a plan component with appropriate scheduling constraints. The scheduling constraints have been obtained from the arcs (see sequenceFlow elements in Fig. 5) pointing to the component being processed. Additionally, for each plan component an appropriate PROforma task has been created. Basically, we have resorted to a decision task for the diverging (exclusive) gateway, and to action tasks for the rest of the components. Within the decision task, a PROforma candidate has been generated for each one of the arcs leaving from the diverging gateway (e.g. action_B and action_C in the case of Fig. 5). In the case of arcs specifying a condition, a rather schematic PROforma argument has been included together with an appropriate PROforma recommendation. The generation of the action tasks is straightforward, except for the actions connected to the diverging gateway, which must include a PROforma precondition.

**Fig. 7:**
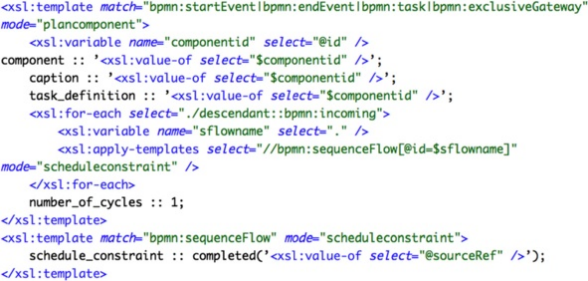
Excerpt of the transformation for generating the PROforma code for the *“exclusive choice—simple merge”* pattern combination: generation of PROforma plan components

As an illustration, Fig. 7 goes here. shows an excerpt of the transformations for the generation of plan components, including the corresponding scheduling constraints.

#### Transformations for workflow control pattern *“persistent trigger”* (#24)

**Fig. 8:**
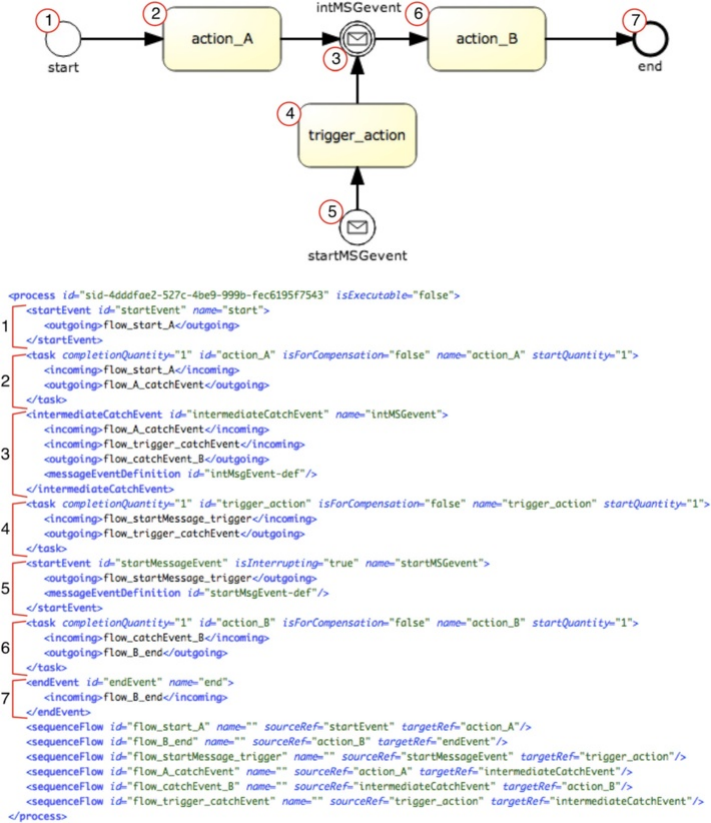
Description of the pattern “persistent trigger” (#24) in BPMN 2.0

Figure 8 shows the BPMN model of the “*persistent trigger”* pattern. This pattern has been defined using message events, more precisely a start message event and an intermediate message event. The former is used to initiate the triggering task depending on a signal from the external environment, similarly to the PROforma implementation in section “Implementation of workflow control patterns for CPGs”. The intermediate message event triggers task action_B, which means that the occurrence of the event serves as an additional constraint for that task.

##### PROforma

**Fig. 9:**
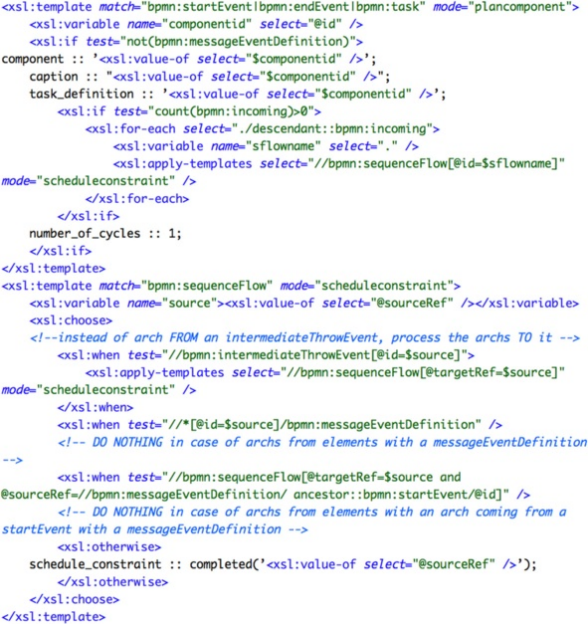
Excerpt of the transformations for generating the PROforma code for the “*persistent trigger”* pattern: generation of PROforma plan components

The transformation is much more complex in this case, due to the different implementation of triggers in BPMN and PROforma. To give an example, a trigger is usually implemented in PROforma through a logical expression in the target task/action, whilst the common representation in BPMN is via an intermediate message event linked to the target task, which is action_B in the case of Fig. 8. To minimize the number of plan components in PROforma, in the transformation process we do not translate this intermediate message event, nor the message event in the triggering task. However, it should be noted that these message events are taken into account in the process, as explained below. Every BPMN task and start/end event is transformed into a PROforma plan component, and an action task is created for it. New scheduling constraints are generated for the action task with a preceding intermediate message event, e.g. action_B in Fig. 8. Otherwise the task would be disconnected from the preceding tasks. For instance, in Fig. 9, although action_A does not directly precede action_B, it is added as a scheduling constraint to the plan component action_B. For the actions without a preceding message event, standard scheduling constraints are added. Besides, an appropriate state trigger (wait_condition) is added to the action task with a preceding intermediate message event. Figure 9 shows a fragment of the transformations for the generation of plan components (and scheduling constraints) in this case, which includes rather intricate XPath expressions.

#### Transformations for workflow control pattern *“interleaved routing”* (#40)

**Fig. 10:**
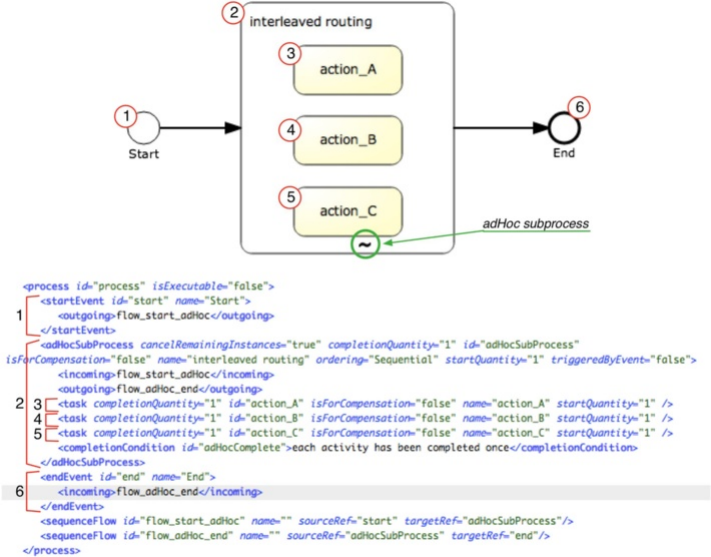
Description of the pattern “interleaved routing” (#40) in BPMN 2.0

Figure 10 shows the graphical model and the XML representation in BPMN 2.0 of three activities that should be interleaved. The model for the *“interleaved routing”* pattern uses an ad hoc sub-process that contains all the activities that must be interleaved and have no predefined sequential order. In this sub-process, the ordering attribute is set to sequential, to allow the execution of only one activity at a time. The element completionCondition defines the condition to determine whether the process has ended. Note that this implementation, which has been proposed by White [33], is an approximation, since it relies on the user/performer to activate each activity exactly once. The implementation uses an ad hoc sub-process, which is a standard BPMN construct.

##### Asbru

**Fig. 11:**
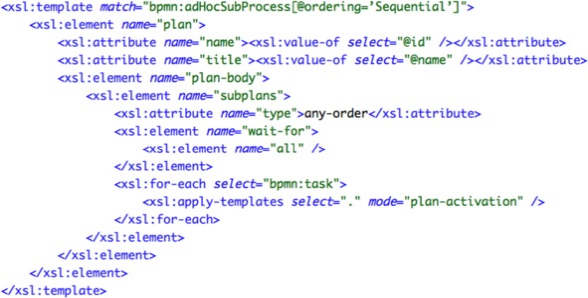
Fragment of the transformations for generating the Asbru code for the *“interleaved routing”* pattern: generation of Asbru plan including the tasks to be interleaved

The transformation to an Asbru model is rather straightforward for this pattern, because Asbru provides a dedicated construct for it. Furthermore, the ad hoc sub-process forms a block around its subtasks in BPMN, which is similar to the Asbru construct. Thus, the ad hoc sub-process (label 2 in Fig. 10) is transformed into a plan containing a subplans element of type “any-order, which executes the subplans sequentially, but without a given order. All activities defined inside the adHocSubProcess element (labels 3 to 5) are also transformed into plan elements in Asbru. Inside the subplans block there are plan-activation elements that point to the plans that have to be activated. Figure 11 shows an excerpt of the transformation.

#### Summary

We provided XSLT transformations for the implemented patterns and we showed that on the one hand BPMN 2.0 can represent the control flow of CPGs and that on the other hand automatic transformations can be applied to obtain executable representations. Note that BPMN 2.0 definitions are rather lax in terms of which elements have to be filled in and how they must be filled in. Therefore, transforming the BPMN 2.0 models into Asbru and PROforma may require post-editing of generated code (e.g. conditions, which are defined only as strings in BPMN 2.0, need to be formalized in Asbru and PROforma) once the transformation has been applied. The full automation of the transformation would require to specialize the model to some degree, as BPMN 2.0 provides some concepts with fewer details. If required this can be overcome by extending the BPMN metamodel to meet the requirements of the target representation, e.g. adding structured conditions that can be translated unambiguously. The BPMN 2.0 metamodel provides a set of extension elements, which allows attaching additional attributes and elements to standard elements, but being still BPMN-compliant.

## Discussion

Back to the research questions we posed in this work, we can draw some lessons. With respect to question (1), we can conclude that workflow control patterns are reasonably suited for the purpose of describing CPG procedural knowledge. However, it should be emphasized that not all the workflow patterns are applicable in the context of CPGs (according to our analysis, only about half of the workflow patterns are applicable). This is mainly due to the fact that CPGs describe processes for decision-support in the context of an individual patient, and not for managing clinical workflow in a wider scope (e.g., involving multiple patients and/or multiple care providers). On the other hand, particular kinds of CPG processes cannot be described in terms of existing workflow patterns and hence new patterns would be required. Notice that these new workflow patterns could be regarded as variations or specializations of existing ones. Having said the above, in our view workflow control patterns can be a valuable tool for the description of CPG processes.

Besides the patterns *“deferred multi-choice”* and *“forced trigger”* suggested by Mulyar et al. [7], we have identified the patterns *“structured discriminator, named task”* and *“structured partial join, named tasks”* (see Table 5 for examples), which are variations of the patterns *“structured discriminator”* and *“structured partial join”*, respectively, with an indication of the name(s) of the task(s) that must be active for synchronization. Such patterns can be found e.g., in Emergency Medicine guidelines, where several tasks are started, but only certain tasks have to be completed to continue with the subsequent task. It should be noted that these patterns can be implemented both in Asbru and PROforma.

**Table 5:** Examples of patterns not covered by the workflow control-flow patterns

New Pattern	Example
Structured discriminator, named task	*Patient should immediately receive oxygen and aspirin. An immediate electrocardiogram should be done and the physician called for as the patient is placed on a cardiac monitor. Intravenous access should be obtained and cardiac markers drawn. … In the critically ill patient whose vitals are compromised (*i.e.*, cardiac arrest, tachyarrhythmias, severe bradycardia, shock or hypotension), the Advanced Cardiac Life Support guideline should be followed* [NB: The critically ill patient is identified through the vital signs coming from the cardiac monitoring] [58]
Structured partial join, named tasks	*Perform: CBC with diff; UA; Cultures—blood and urine; (Consider wait on LP); Stool for WBC and culture (if diarrhea); Chest radiograph (if respiratory signs) If all low risk clinical and laboratory criteria met (see Table* 2 *)* [NB: In urinalysis, <10 WBC/hpf; In CBC, WBC 5,000 to 15,000/mm3; etc.; cultures for blood, urine, and stool are taken, but care is continued without waiting for results] *then consider outpatient management or admission for observation* [57]

With respect to question (2), we have shown that the workflow patterns that are relevant in the context of CPGs can be implemented in two different CPG languages to a big extent. Here it should be noticed that out of the 22 relevant patterns only 1 pattern in Asbru and 3 patterns in PROforma were not implementable. However, it would be possible to model the corresponding part of the CPG through programming workarounds.

Lastly, regarding question (3), we have shown that individual patterns can be transformed from a process specification (BPMN 2.0) to different executable implementations (Asbru and PROforma). Although real-world models will be more complex and contain multiple and varied patterns, a pattern-based transformation can form the basis of a framework for transforming whole CPG models. Utilizing BPMN 2.0 is a useful approach to provide a domain-independent and neutral process specification, which can serve as a basis for different final executable process implementations.

The work described in this article presents some limitations. One is the reduced size of the CPG sample used in the suitability analysis. Being aware of this, the patterns for which no supporting example was found in the CPG sample have been considered as potentially suitable. Notice that only the patterns about which there exist justified doubts have been disregarded. Another limitation is that we have not considered patterns for important aspects of CPGs other than procedural ones, such as data and evidence. Finally, it should be noticed that the suitability analysis has been carried out by the authors of the paper, who are computer scientists experienced in CPG modelling but without a medical training. In our view, a similar outcome would have been obtained if experts with a different background (e.g. clinicians), but also experienced in CPG modeling, had participated in the study.

With respect to the pattern and transformation-based approach we propose, preliminary experiments confirm that real-world CPGs can be fully modeled with a subset of the patterns we identified (e.g. in a prostate cancer CPG we only used patterns #1, #4-#5, #6-#7, #21, and #40, all of them multiple times). However, for the moment we have not evaluated the validity and effectiveness of the approach in a realistic setting, with the involvement of both clinical experts and knowledge engineers. An important issue for the applicability of our approach is the recognition of fragments representing a pattern, both in CPG texts and in BPMN 2.0 models. For the latter, we have obtained significant results based on algorithmic approaches for the recognition of basic workflow patterns in BPMN 2.0 models of arbitrary size and complexity [49]. On another level, the verification and validation issues related to our transformation-based view have been left out from the approach, since advanced techniques specific to the Asbru and PROforma languages are already available [50, 51].

## Conclusions

Using patterns for describing frequent structures is a well-known method that has been applied to the perspective of control flow in the domain of process modeling. We propose a pattern and transformation-based approach for the development of CPG models. The identification of adequate patterns and the implementation of transformations to convert patterns from a process specification into different executable implementations are the first necessary steps for our approach.

There are several contributions in this article. One contribution is the analysis of workflow control patterns from a different perspective, taking into account their adequacy for the representation of CPG procedural knowledge, as opposed to previous studies. Moreover, as a by-product of this analysis we have identified a number of additional patterns that can be considered in future investigations. Another contribution is the development of a series of pattern-based transformations that can be used to semi-automatically convert the BPMN 2.0 specification of CPG fragments into the Asbru and PROforma languages. Finally, a key contribution is the pattern and transformation-based approach itself. Preliminary experiments suggest that such an approach can form the basis of a valid framework for the authoring of CPG models.

In the future we plan to conduct an experiment to evaluate the validity and effectiveness of our approach in a more realistic setting. Additionally, we intend to analyze other aspects which are part of CPGs, such as data and time, for which corresponding patterns have been defined [52, 53]. We will analyze these patterns with the purpose of integrating them in a comprehensive framework to assist the modeling of CPGs.

## Abbreviation

BPM: Business Process Management
BPMN: Business Process Model and Notation
CPG: clinical practice guideline
GEM: Guideline Elements Model
GLIF: GuideLine Interchange Format
MHB: Many-Headed Bridge
MI: multiple instances
OMG: Object Management Group
SAGE: standards-based Shareable Active Guideline Environment
UMLS: Unified Medical Language System
XML: eXtended Markup Language
XSL: eXtended Stylesheet Language
XSLT: XSL Transformation

## Footnotes

1 In this paper, the terms “workflow control pattern” and “workflow pattern” are used indistinctively to refer to the workflow control-flow patterns by Russell et al. [15].
